# Two-year nighttime blood pressure changes after radiofrequency renal denervation: pooled results from the SPYRAL HTN trials

**DOI:** 10.1038/s41440-025-02186-z

**Published:** 2025-04-02

**Authors:** Kazuomi Kario, David E. Kandzari, Felix Mahfoud, Michael A. Weber, Roland E. Schmieder, Konstantinos Tsioufis, Minglei Liu, Michael Böhm, Raymond R. Townsend

**Affiliations:** 1 Department of Cardiovascular Medicine, Jichi Medical University School of Medicine, Tochigi, Japan; 2 Piedmont Heart Institute, Atlanta, GA USA; 3 Cardiovascular Research Institute Basel (CRIB), University Heart Center, University Hospital Basel, Basel, Switzerland; 4 Department of Cardiology, University Hospital Basel, Basel, Switzerland; 5 SUNY Downstate College of Medicine, New York, NY USA; 6 University Hospital Erlangen, Friedrich Alexander University Erlangen/Nurnberg, Nurnberg, Germany; 7 National and Kapodistrian University of Athens Hippocratio Hospital, Athens, Greece; 8 Medtronic Inc, Santa Rosa, CA USA; 9 Universitätsklinikum des Saarlandes, Saarland University, Homburg, Germany; 10 Perelman School of Medicine, University of Pennsylvania, Philadelphia, PA USA

**Keywords:** Renal denervation, Nocturnal hypertension, Cardiovascular risk

## Abstract

Elevated nighttime blood pressure (BP) and abnormal circadian dipping patterns are associated with advanced age and coexisting illnesses and are attributed to autonomic dysfunction. Radiofrequency renal denervation (RF RDN) effectively lowers BP throughout 24 h and thus may provide an effective antihypertensive therapeutic option. This analysis assesses the effects of RDN on nocturnal hypertension with different dipper patterns defined by nighttime/daytime BP ratio (i.e. dippers, non-dippers, risers) through 2 years in patients randomized to RDN from the SPYRAL HTN-OFF MED and -ON MED trials. Office and 24-h ambulatory BP, were also evaluated in patients stratified by age, obstructive sleep apnea (OSA), type 2 diabetes mellitus (T2DM) and chronic kidney disease (CKD). Among 388 patients, the baseline nighttime systolic BP (SBP) was 139.3 ± 11.3 mmHg. Patients with a riser pattern had the highest baseline nighttime SBP (152.7 ± 8.0 mmHg). At 2 years, patients experienced a significant reduction from baseline (*p* < 0.0001) in nighttime (−12.0 ± 17.1 mmHg), morning (−14.8 ± 20.0 mmHg), daytime (−13.8 ± 14.7 mmHg), and 24-h SBP (−13.4 ± 14.2 mmHg). The greatest reduction in SBP was in risers at nighttime (−23.7 ± 14.3 mmHg). RDN was equally effective in lowering nighttime BP in patients ≥65 years old or with OSA, CKD, or T2DM. In this pooled dataset of RF RDN patients, clinically meaningful reductions in BP over a 24-h period were observed through 2 years irrespective of dipping status. RF RDN may reduce the risk of cardiovascular outcomes in patients with uncontrolled hypertension, especially in those with elevated nighttime BP who may be the most challenging to treat.

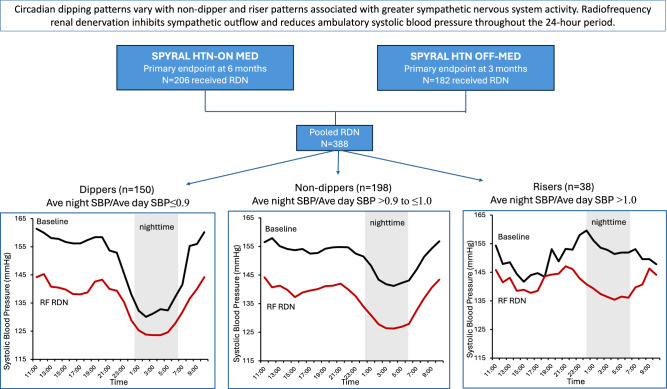

## Introduction

Elevated nighttime blood pressure (BP), or nocturnal hypertension and an abnormal circadian dipping pattern (same or higher nighttime than daytime BP) are both associated with increased cardiovascular morbidity and mortality [1, 2]. The strongest relative association between increased BP and cardiovascular risk occurs at nighttime compared with morning or daytime BP [3]. Nocturnal hypertension and an abnormal circadian dipping pattern are attributed to increased autonomic nervous system activity and are also associated with older age, diabetes mellitus (DM), chronic kidney disease (CKD), and obstructive sleep apnea (OSA) [4].

Recently, randomized sham-controlled trials have demonstrated the safety and efficacy of radiofrequency renal denervation (RF RDN) to lower BP, in both the presence and absence of concomitant antihypertensive pharmacotherapy [5, 6]. RF RDN also has demonstrated a consistent 24-h, “always on” effect without dependence on patient’s adherence to drug and lifestyle interventions [6]. Therefore, RF RDN may offer a unique additional BP lowering effect for patients with nocturnal hypertension.

Previously, we reported significantly greater nighttime BP reductions after 3 years following RF RDN compared with sham in patients with apparent resistant hypertension prescribed up to 3 antihypertensive drugs in the SPYRAL HTN-ON MED Pilot study [7]. In this analysis, we investigated the long-term effect of RF RDN on nighttime BP and different circadian dipping patterns from a larger pool of patients in the SPYRAL HTN trials including patients stratified by age and status of CKD, Type-2 DM (T2DM), and OSA.

## Methods

### Study design and patient population

The SPYRAL HTN-OFF MED Pivotal and -ON MED studies were multicenter, international, prospective, blinded, randomized, sham-controlled trials designed to assess the safety and efficacy of RF RDN in uncontrolled hypertensive patients off and on prescribed antihypertensive drugs, respectively, and the designs of which have been previously published [8]. Patients were required to be between 20 and 80 years of age with uncontrolled HTN, defined as office systolic BP (SBP) ≥ 150 mmHg and <180 mmHg, office diastolic BP (DBP) ≥ 90 mmHg, and mean 24-h ambulatory SBP ≥ 140 mmHg and <170 mmHg. Patients in the OFF MED trial were required to discontinue antihypertensive drugs prior to randomization and remain off through 3 months post randomization, whereas patients in ON MED were required to be on a stable regimen of 1–3 antihypertensive drugs for up to 6 months post-randomization. 182 patients from OFF MED Pivotal and 206 patients from ON MED were randomized and underwent RF RDN, resulting in the pooled RF RDN cohort of 388 patients. All patients provided written informed consent. The protocol was approved by all local ethics committees. The studies were designed in accordance with the Declaration of Helsinki.

### Procedure

The details of the RDN procedures have been previously described [5, 8–10]. Briefly, the RDN procedure was conducted using the multielectrode Symplicity Spyral^™^ RDN catheter and Symplicity G3™ RDN radiofrequency generator (Medtronic Plc), providing circumferential ablation treatments of all renal arteries and branch vessels between 3 and 8 mm in diameter. Cases were performed by experienced proceduralists and proctored according to specific treatment plans. Angiography was performed throughout the procedure to verify anatomy and catheter placement.

### Follow-up

Both office or clinic BP and 24-h ambulatory BP were measured at baseline and at 3, 6, 12, and 24 months after RF RDN. Office BP was measured before witnessed pill-intake and 24-h ambulatory BP commenced after witness pill-intake per study design. Antihypertensive drug and dosage changes were prohibited through 6 months in the ON MED trial, unless pre-specified blood pressure criteria were met. Antihypertensive drugs could be prescribed in the -OFF MED trial for patients with an office SBP ≥ 140 mmHg after the 3-month visit. The number of antihypertensive drugs were estimated based on drug testing if available and prescribed otherwise.

### Definitions

Nocturnal hypertension was defined as nighttime BP > 120/70 mmHg as per the most recent European Society of Hypertension guideline on hypertension management [11]. Nighttime BP was measured between 1 and 6 AM, morning BP between 7 and 9 AM and daytime BP between 9 AM and 9 PM [7]. Dipping patterns were categorized by their ratio of nighttime/daytime systolic BP: dippers ≤0.9, non-dippers >0.9 to ≤1.0 and risers (i.e. reverse dippers) >1.0 [12]. For purposes of determining dipping patterns the nighttime period was defined as 10 PM to 7 AM and daytime was defined as 7 AM to 10 PM. The same ratios were used to define 24-h heart rate dipping patterns. Peak SBP, the average of three, maximum SBP measurements, was determined for the nighttime, morning, daytime, and average 24-h ambulatory time periods.

### Statistical analysis

All patients randomized to receive RF RDN on the ON and OFF MED trials were included in this analysis. Baseline characteristics of all pooled patients and patients stratified by dipping patterns were compared across groups. BP changes from baseline through 2 years after RDN and peak SBP at different times of the day were also evaluated by dipping patterns. Categorical measures were expressed as percentages and compared using chi-square tests. Continuous measures were expressed as mean ± standard deviation (SD). Changes from baseline was evaluated using paired t-test and between group comparisons were performed using analysis of variance (ANOVA) for baseline measurements, or analysis of covariance (ANCOVA) for changes from baseline adjusted for baseline values. Statistical significance was established as *p* < 0.05. Statistical analyses were performed using SAS for Windows 9.4 (SAS Institute, Research Triangle, NC).

## Results

The proportion of patients with nocturnal hypertension at baseline was 96.1%. Two years after RF RDN, the proportion of patients with nocturnal hypertension was reduced by 27.4% from baseline (*p* < 0.0001). The number of antihypertensive drugs increased from 1.55 ± 1.06 at 6 months (when OFF MED patients could also be prescribed antihypertensive pharmacotherapy) to 1.92 ± 1.18 at 2 years in the overall pooled cohort (Supplementary Table 1).

**Fig. 1:**
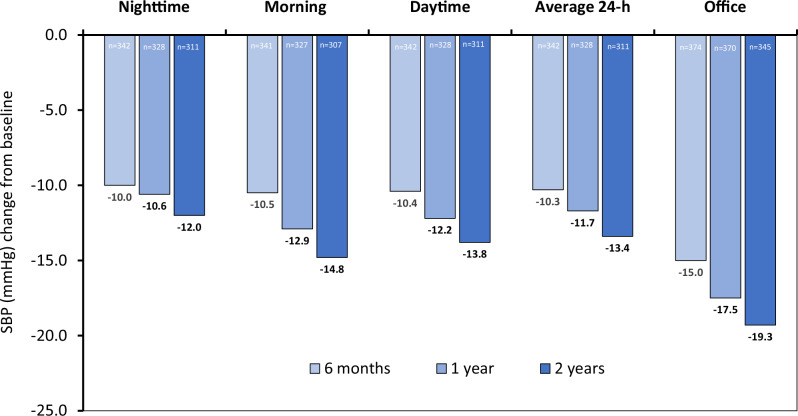
Mean systolic BP changes through 2 years after radiofrequency RDN. There was a progressive reduction in mean systolic BP through 2 years follow-up, at all times of the day. All BP changes compared to baseline were statistically significant *p* < 0.05

**Fig. 2:**
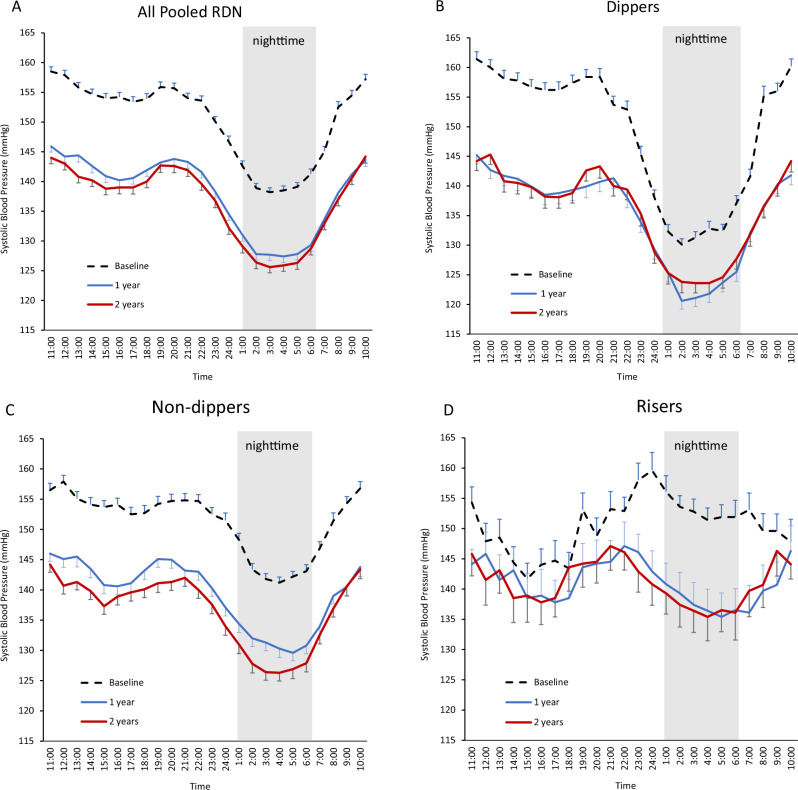
24-h systolic BP at baseline, 1 year and 2 years. The overall cohort (**A**), dipper (**B**) and non-dipper (**C**) patients followed the typical circadian cycle of BP change at all time points. The riser group (**D**) experienced significant nighttime reductions in SBP with dampening of the nighttime rise in SBP

Three hundred eighty-eight patients who underwent RDN were evaluated. At baseline, patients were 54 ± 10 years old, 26.8% women, BMI 31.3 ± 6.0 kg/m^2^, 45.1% had hypertension >10 years, 7.7% had T2DM, 9.8% had OSA, and 5.4% had CKD defined by an estimated glomerular filtration rate (eGFR) < 60 ml/min/1.73 m^2^ (Table 1). In the overall cohort, the baseline nighttime SBP was 139.3 ± 11.3 mmHg, morning SBP was 153.6 ± 15.0 mmHg, daytime SBP was 155.6 ± 8.5 mmHg, average 24-h ambulatory SBP was 150.3 ± 7.4 mmHg and office SBP was 162.9 ± 7.7 mmHg. The diastolic measures are also shown in Table 1. At 2 years after RF RDN, the overall cohort had significant reductions in SBP from baseline (*p* < 0.0001): nighttime SBP (−12.0 ± 17.1 mmHg), morning SBP (−14.8 ± 20.0 mmHg), daytime SBP (−13.8 ± 14.7 mmHg), 24-h SBP (−13.4 ± 14.2 mmHg), and office SBP (−19.3 ± 15.6 mmHg) (Fig. 1). The mean 24-h SBP followed the typical rise and fall of BP throughout the circadian cycle (Fig. 2A).

**Table 1:** Baseline patient characteristics and hemodynamic measure

Demographics	All (*n* = 388)	Dippers (*n* = 150)	Non-Dippers (*n* = 198)	Risers (*n* = 38)	*P* value*
Age (yrs)	54.0 ± 9.9	54.8 ± 9.3	53.5 ± 9.9	54.0 ± 12.4	0.49
Female	26.8%	29.3%	27.8%	10.5%	0.06
Body mass index (kg/m^2^)	31.3 ± 6.0	30.5 ± 5.5	31.9 ± 6.2	31.3 ± 6.7	0.10
Type 2 diabetes mellitus	7.7%	7.3%	8.1%	7.9%	0.97
Coronary artery disease	2.8%	2.7%	3.0%	2.6%	0.98
Myocardial infarction	1.0%	1.3%	0.5%	2.6%	0.45
Heart failure	0.0%	0.0%	0.0%	0.0%	
Left ventricular hypertrophy	6.2%	7.3%	5.6%	5.3%	0.77
Stroke	0.3%	0.0%	0.5%	0.0%	0.62
Atrial fibrillation	1.5%	3.3%	0.5%	0.0%	0.08
Obstructive sleep apnea	9.8%	9.3%	9.6%	13.2%	0.77
CKD (eGFR >45–<60 ml/min/1.73 m^2^)	5.4%	4.0%	6.1%	7.9%	0.55
Hyperthyroidism	0.3%	0.0%	0.0%	2.6%	0.01
Smoking -current	16.2%	14.0%	17.2%	21.1%	0.52
Nighttime (1am–6am)					
Systolic (mmHg)	139.3 ± 11.3	131.8 ± 9.4	142.4 ± 9.2	152.7 ± 8.0	<0.001
Diastolic (mmHg)	87.9 ± 10.3	82.3 ± 9.3	90.5 ± 9.1	95.9 ± 9.5	<0.001
Morning (7am–9am)					
Systolic (mmHg)	153.6 ± 15.0	155.3 ± 14.6	153.2 ± 15.2	149.3 ± 15.2	0.06
Diastolic (mmHg)	100.5 ± 11.3	100.7 ± 11.0	100.7 ± 11.6	98.4 ± 11.5	0.50
Daytime (9am–9pm)					
Systolic (mmHg)	155.6 ± 8.5	158.5 ± 7.8	154.9 ± 8.1	148.0 ± 7.4	<0.001
Diastolic (mmHg)	101.8 ± 8.0	102.5 ± 8.2	101.9 ± 7.9	98.0 ± 6.4	0.006
Average 24-h ambulatory					
Systolic (mmHg)	150.3 ± 7.4	149.0 ± 7.1	151.2 ± 7.6	150.8 ± 7.2	0.03
Diastolic (mmHg)	97.1 ± 7.7	95.3 ± 7.7	98.3 ± 7.7	97.5 ± 6.7	0.002
Heart rate (bpm)	74.5 ± 10.5	73.4 ± 10.4	75.2 ± 10.4	74.8 ± 10.6	0.31
Pulse pressure (mmHg)	53.3 ± 7.6	53.7 ± 7.9	52.9 ± 7.4	53.3 ± 7.4	0.61
Office/clinic					
Systolic (mmHg)	162.9 ± 7.7	163.6 ± 7.6	162.5 ± 7.8	161.9 ± 7.7	0.32
Diastolic (mmHg)	101.1 ± 7.0	100.4 ± 6.7	101.7 ± 7.2	100.4 ± 7.1	0.19
Heart rate (bpm)	73.4 ± 10.9	72.5 ± 10.7	73.9 ± 11.3	73.8 ± 9.6	0.47
Pulse pressure (mmHg)	61.7 ± 9.3	63.2 ± 9.7	60.8 ± 9.0	61.4 ± 9.0	0.06

Data is shown as % or *n* ± SD

*P values represent comparison between dippers, non-dippers and risers

At baseline, patients were categorized as dippers (*n* = 150; 38.9%), non-dippers (*n* = 198; 51.3%) and risers (*n* = 38; 9.8%), respectively. Risers had the highest baseline nighttime SBP (152.7 ± 8.0 mmHg) and the lowest daytime SBP (148.0 ± 7.4 mmHg) compared to other dipping pattern groups (Table 1). Again, the typical 24-h circadian cycle pattern of BP change was observed at baseline in dippers (Fig. 2B) as well as in non-dippers (Fig. 2C). However, the riser group featured a characteristic dampening of the nighttime BP reduction (Fig. 2D). The riser group included relatively fewer women, and more patients with history of myocardial infarction, CKD, or OSA, but none of these differences were statistically significant between groups (Table 1).

**Fig. 3:**
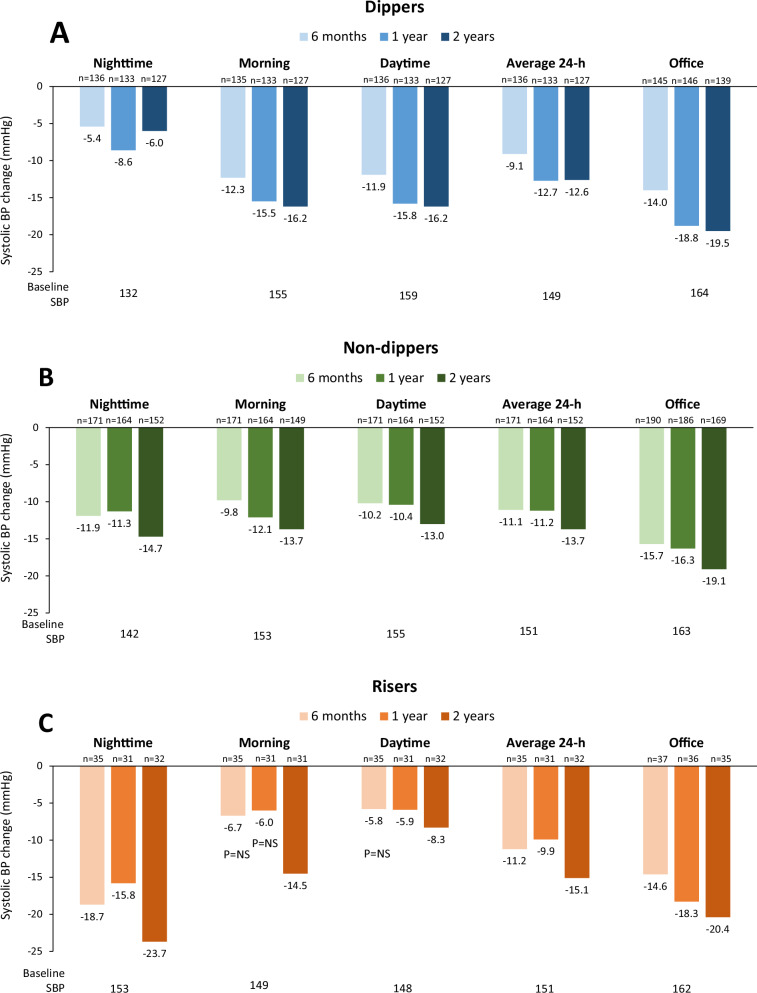
Mean systolic BP changes through 2 years after radiofrequency RDN by baseline dipping patterns. There was generally a progressive reduction in mean systolic BP through 2 yrs follow-up, at all times of the day. **A** Dippers, **B** non-dippers, **C** risers. All BP changes compared to baseline were statistically significant *p* < 0.05 except 6-month morning and day SBP and 1-year morning SBP change in risers

The greatest magnitude of SBP reduction at 2 years was observed for nighttime SBP in the riser group (−23.7 ± 14.3 mmHg; Fig. 3C). Similarly, the greatest magnitude of DBP reduction was observed for nighttime DBP at 2 years in the riser group (−15.9 ± 8.1 mmHg; Supplementary Fig. 1C). However, in dippers and non-dippers, the greatest SBP reduction from baseline to 2 years was in the office SBP (dippers: −19.5 ± 15.2 mmHg, non-dippers: −19.1 ± 15.4 mmHg), compared with prespecified day and night intervals (Fig. 3A, B).

The mean 24-h heart rate at baseline was 74.5 ± 10.5 bpm for the overall cohort and was similar across the SBP dipping pattern groups (Table 1). In the overall cohort there were 23/386 (6%) heart rate risers of which 4 patients were also in the SBP riser group (Supplementary Table 2). At 2 years, the 24-h heart rate dropped significantly in the SBP dipper group (73.4 ± 10.4 bpm to 71.2 ± 9.7 bpm, *p* = 0.006), but there was no significant change in heart rate in the SBP non-dipper (75.2 ± 10.4 bpm to 73.6 ± 10.8 bpm, *p* = 0.072) and SBP riser (74.8 ± 10.6 bpm to 75.0 ± 10.3 bpm, *p* = 0.274) groups (Supplementary Table 3).

The peak SBP was also evaluated in the overall cohort, and by dipper patterns. The highest peak SBP in the overall cohort was peak 24-h SBP (184.4 ± 13.3 mmHg), followed by peak daytime SBP (180.7 ± 13.0 mmHg), peak morning SBP (157.0 ± 15.4 mmHg) and peak nighttime SBP (151.5 ± 13.7 mmHg). For risers, the baseline peak nighttime SBP was 165.4 ± 10.3 mmHg, which changed by −23.1 ± 16.7 mmHg after RDN at 2 years. Significant reductions in the peak SBP from baseline in other dipping patterns are shown in Table 2.

**Table 2:** Peak systolic blood pressure at baseline and changes 2 years after RDN

Baseline peak systolic BP	All (*n* = 388)	Dippers (*n* = 150)	Non-Dippers (*n* = 198)	Risers (*n* = 38)	*P* value*
Nighttime (1 am–6 am)	151.5 ± 13.7	144.0 ± 12.4	154.5 ± 11.8	165.4 ± 10.3	<0.001
Morning (7 am–9 am)	157.0 ± 15.4	158.5 ± 15.3	156.5 ± 15.3	153.4 ± 16.3	0.16
Daytime (9 am–9 pm)	180.7 ± 13.0	183.5 ± 12.4	179.7 ± 12.8	175.1 ± 14.1	<0.001
Average 24-h ambulatory	184.4 ± 13.3	185.7 ± 12.9	183.7 ± 13.4	183.0 ± 14.2	0.29
Changes in peak systolic BP at 2 years					
Nighttime (1 am–6 am)	−12.3 ± 19.7	−6.0 ± 18.6	−15.3 ± 19.6	−23.1 ± 16.7	0.51
Morning (7 am−9 am)	−14.5 ± 21.1	−15.6 ± 23.4	−13.5 ± 18.9	−14.8 ± 21.3	0.98
Daytime (9 am–9 pm)	−15.2 ± 19.9	−16.5 ± 19.8	−15.0 ± 17.9	−11.0 ± 28.0	0.63
Average 24-h ambulatory	−15.4 ± 20.7	−15.7 ± 20.6	−15.1 ± 18.6	−15.7 ± 29.2	0.75

^*^P values represent comparison between dippers, non-dippers and risers. Changes at 2 years are adjusted for baseline values. Peak values represent the average of the three highest measurements within the time period specified

### Subgroup outcomes

**Fig. 4:**
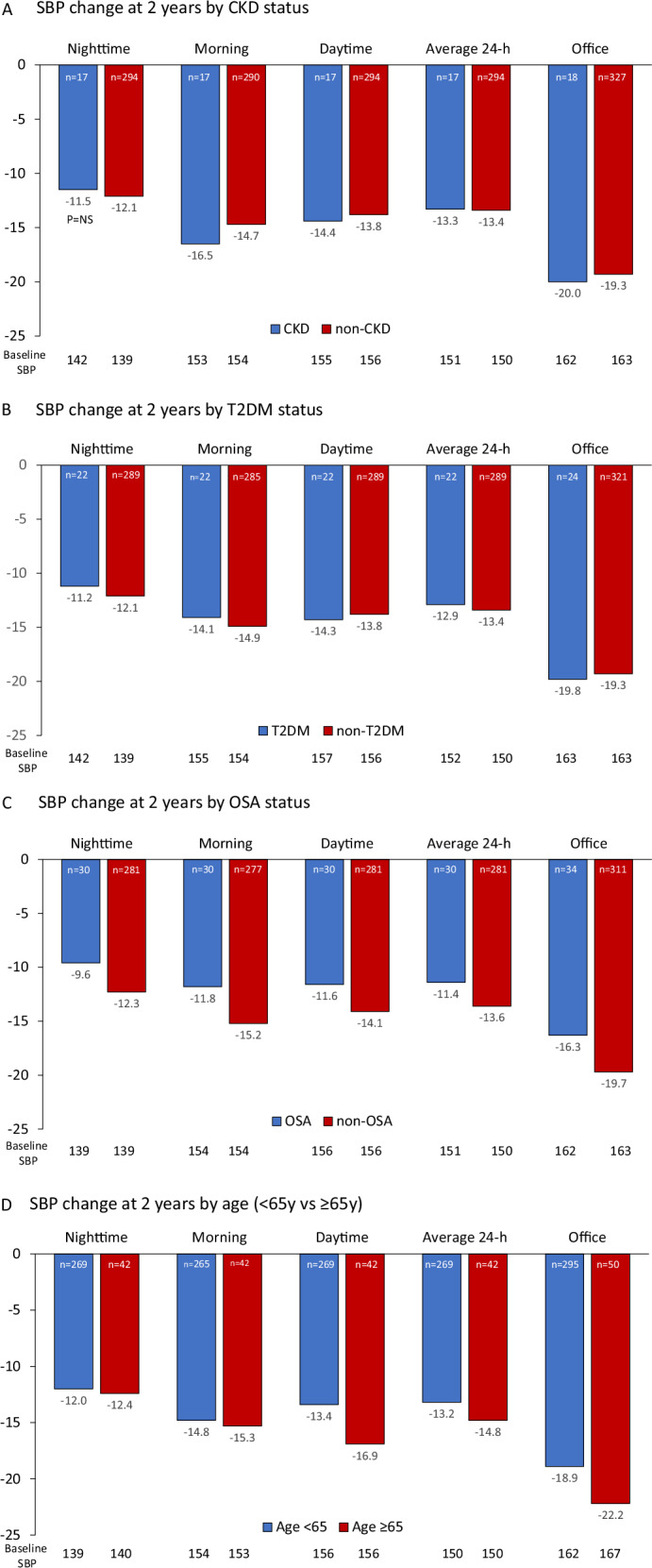
Change in systolic BP at 2 years for subgroups. Significant SBP reductions were observed at 2 years post RF RDN in patients with CKD (**A**), T2DM (**B**), OSA (**C**) and in patients ≥65 years of age (**D**). There were no significant differences between patients with and without CKD, T2DM or OSA or between younger vs older patients. All SBP changes from baseline are significant (*p* < 0.05) expect the nighttime drop for patients with CKD

Patients with CKD experienced clinically meaningful reductions in office, 24-h, morning, daytime, and nighttime SBP at 2 years that did not significantly differ from SBP reductions in non-CKD patients (office SBP −19.3 ± 15.2 mmHg; 24-h SBP −13.4 ± 14.1 mmHg) (Fig. 4A). The nighttime SBP change from baseline was not statistically significant in the CKD group. At all specified time intervals significant SBP reductions were observed in patients with T2DM (office SBP −19.8 ± 13.0 mmHg; 24-h SBP −12.9 ± 13.2 mmHg) and OSA (office SBP −16.3 ± 17.2 mmHg; 24-h SBP −11.4 ± 19.4 mmHg) (Fig. 4B, C). Patients ≥65 years old had similar, significant reductions in SBP across 24 h (SBP −14.8 ± 11.1 mmHg) and in office (SBP −22.2 ± 17.8 mmHg) compared with younger patients (Fig. 4D). There is no significant difference in the distribution of baseline dipping patterns between patients with and without CKD, T2DM, and OSA or in older vs younger patients (Supplementary Table 4).

## Discussion

In this post-hoc analysis of the SPYRAL HTN OFF and ON MED trials, 96.1% of the patients had nocturnal hypertension and more than half the patients had an abnormal circadian dipping pattern (non-dippers and risers; 60.8%). Two years following RF RDN, all three groups had significantly lower nighttime BP compared to baseline, especially in risers who had the highest baseline nighttime BP. In addition to nighttime BP reductions, there was a constant, 24-h “always on” effect of RDN reducing BP at other times of the day, which generally progressed over two years of follow-up. RF RDN was equally effective in patients with and without OSA, T2DM, and CKD, as well as in patients within both age categories.

The importance of out-of-office BP monitoring has been highlighted by current guidelines and is increasingly utilized in clinical and research settings [13]. High nighttime BP is more strongly associated with cardiovascular death compared to high BP at other times of the day^1^, and nighttime BP assessment is recommended by the most recent European Society of Hypertension guideline for effective management of hypertension [14]. Nocturnal hypertension has been shown to correlate with greater sympathetic nerve activation compared with daytime sympathetic nerve activity [15]. Furthermore, risers have 10–15 mmHg higher nighttime SBP compared to other dipping patterns [16], consistent with our findings. Risers are thought to have severely abnormal circadian variation, which may be related to increased activation of the sympathetic neural pathway as suggested by measurements of muscle sympathetic nerve activity (MSNA) in normotensive and hypertensive patients. Comparison of MSNA levels between dippers, extreme dippers, non-dippers and risers revealed that risers had significantly higher sympathetic tonus activation than all other hypertensive patients (*p* < 0.05) [17], suggesting that there is an inverse relationship between elevated sympathetic activation and the degree of nighttime BP reduction following RDN.

Despite the importance of controlling nocturnal hypertension, increasing pharmacotherapy has limited effects on the nighttime BP, as antihypertensive drugs are often taken once daily in the morning, and have waning pharmacodynamics after drug-clearance by night [18]. A study of over 2700 hypertensive patients, found that in patients prescribed 3 or more antihypertensive medications, nighttime BP was uncontrolled in 46.5% of patients [19]. This observation highlights the importance of monitoring BP throughout the 24 circadian cycle and the potential value of a non-pharmacologic interventional option such as RF RDN for control of nocturnal hypertension in patients who may be nonadherent to complex pharmacologic regimens [11, 14].

A riser BP pattern is associated with organ damage [20], dementia [21, 22], poor cardiovascular prognosis [2], and is particularly associated with heart failure with preserved ejection fraction relative to heart failure with reduced ejection fractions [23]. Interestingly, the non-dipping pattern, including risers, is also associated with a higher number of baseline antihypertensive drugs, and is not significantly affected by different timing of drug-intake [16]. In our analysis non-dipping patterns were not differentiated by office BP values compared with the other nighttime BP groupings (Table 1).

A riser heart rate pattern is also associated with substantially higher risk for cardiovascular events including stroke and heart failure as demonstrated by an analysis of over 6000 patients enrolled on The Japan Ambulatory Blood Pressure Monitoring Prospective (JAMP) study [24]. The JAMP study found that the highest risk for cardiovascular events was in the patients that had a riser pattern of both SBP and heart rate. However, there was not a strong correlation between SBP and heart rate dipping patterns, consistent with our data. Only 10.5% of patients with a SBP riser pattern were also heart rate risers. These data suggest differing mechanisms may underlay dipping status for SBP and heart rate.

Similarly, peak SBP has been associated with higher risk for organ damage and stroke independent of other BP variability parameters such as coefficient of variation and average real variability [25–27]. In the Japan Morning Surge-Home Blood Pressure (J-HOP) study with >6 years follow-up using home BP monitors [27], the pathological threshold for the highest risk of stroke was 176 mmHg. In this study, the baseline peak 24-h ambulatory SBP and the peak daytime SBP in the overall cohort exceeded this pathological threshold (>180 mmHg). This was particularly true within the dipper subgroup, who have the most favorable circadian dipping pattern associated with the lowest cardiovascular risk compared to other dipping patterns. Two years after RDN, the peak SBP in both 24-h and daytime were lowered below this pathological threshold to <170 mmHg. Recently, the J-HOP study also demonstrated a significant association between the peak nighttime home SBP and high risk of stroke [28], with a pathological threshold of 136 mmHg. In this study, we observed a high baseline peak nighttime SBP of >140 mmHg, even within dippers with the lowest nighttime baseline SBP compared to other dipping patterns. Therefore, patients enrolled in this study reflect a high-risk population, including the dippers who are conventionally thought to have the lowest cardiovascular risk, and all may benefit by the peak BP lowering effect of RDN in addition to pharmacotherapy.

The seminal SPRINT (Systolic Blood Pressure Intervention Trial) ABPM substudy [29] showed that patients prescribed standard antihypertensive therapy (average 2 drugs) compared to patients prescribed intensive therapy (average 3 drugs) had a nighttime SBP difference of 9.8 mmHg favoring the intensive antihypertensive therapy at 27 months follow-up. Although a direct comparison is not possible due to different design and objective of the studies, at 2 years, this pooled analysis of the SPYRAL HTN OFF and ON MED trials showed a nighttime SBP difference of 12.0 mmHg after patients had RF RDN. Furthermore, we previously showed a statistically significant difference in the nighttime SBP favoring RDN compared to sham control, from the SPYRAL HTN-ON MED trial [5, 7], suggesting a unique RDN effect on nighttime BP compared to pharmacological treatment alone.

We also examined overall and nighttime BP changes in patients ≥65 years old and in subgroups of patients with comorbidities including T2DM, OSA, and CKD that may further increase an individual’s risk for cardiovascular disease, stroke, and kidney damage in combination with nocturnal hypertension [30–32]. An estimated 80% of patients with CKD have elevated BP which is often difficult to control [33]. High sympathetic activity is also commonly associated with CKD suggesting that RDN may be especially beneficial in this population [31]. Our study of pooled RDN data from 2 Spyral HTN trials is consistent with previous analyses of RF RDN in the Global SYMPLICITY registry (GSR) that reported 24 h SBP change from baseline of −7.2 ± 21.2 mmHg at 2 years and −9.2 ± 19.5 at 3 years in patients with CKD [34].

In individuals with diabetes mellitus, hypertension is associated with a 72% increased risk for all-cause death and a 57% increased risk for a cardiovascular event [35]. The GSR has also reported the blood pressure lowering effect of RF RDN in high-risk subgroups including CKD, T2DM and patients age ≥65 years and found similar significant reductions in 24-h SBP [36]. A further analysis of this all-comer registry estimated clinical event reductions in these high-risk groups based on observed reductions in SBP from baseline and evidence from published meta-regression analysis. Results indicated that RF RDN may lower the risk for major adverse cardiovascular events by almost one-third within 3 years [37].

Current evidence indicates a bidirectional, causal relationship between OSA and hypertension with a prevalence of OSA in hypertensive patients ranging from 30% to 50% [38]. OSA and nighttime hypertension are both associated with increased sympathetic drive with non-dippers and risers more likely to have OSA [38]. A smaller randomized proof-of-concept trial examined the effect of RDN in patients with OSA and reported significant reductions in office and 24-h ambulatory BP compared to a non-treated control group [39]. We found similar significant reductions in BP in patients reporting OSA in the current pooled RDN population including in nighttime BP. However, the number of patients reporting OSA was only 38 (9.8%) with 5 (13.2%) in the riser group prohibiting analysis across circadian dipping patterns. Further research with a larger number of patients with OSA and hypertension is indicated to better understand the relationship of non-dipping patterns in this population.

### Limitations

This was a post-hoc analysis of pooled data obtained from patients receiving RF RDN on two randomized controlled trials with different protocols regarding antihypertensive drug use, and with the primary endpoints evaluated at different timepoints. Each of these trials allowed antihypertensive medication changes after the primary endpoint was reached which could impact nighttime BP measures and dipping status. Different patient behaviors between the RDN and sham control groups regarding drug adherence and lack of data from sham control patients crossing over to receive RDN before 2 years follow-up did not allow for direct comparison between the RDN and sham control groups, hence this analysis excluded the sham group.

## Conclusions

In this post-hoc analysis of the SPYRAL HTN OFF and ON MED trials in patients with uncontrolled hypertension, most of the patients had nocturnal hypertension and a significant proportion had an abnormal circadian dipping pattern. Two years after RF RDN, significant nighttime BP reductions were observed in addition to other times of the day including in older patients and patients with OSA, T2DM or CKD at baseline. Clinically meaning BP reductions were observed irrespective of dipper status although the largest peak SBP reduction was observed in patients with a riser pattern at baseline. These results suggest that patients with high nighttime BP, especially risers who are at higher risk for stroke and other associated consequences of hypertension, may particularly benefit from RF RDN.

## Supplementary information

Supplementary Material

## References

[1] Staplin N, de la Sierra A, Ruilope LM, Emberson JR, Vinyoles E, Gorostidi M, et al. Relationship between clinic and ambulatory blood pressure and mortality: an observational cohort study in 59 124 patients. Lancet. 2023;401:2041–50. 37156250 10.1016/S0140-6736(23)00733-X

[2] Kario K, Hoshide S, Mizuno H, Kabutoya T, Nishizawa M, Yoshida T, et al. Nighttime Blood Pressure Phenotype and Cardiovascular Prognosis: Practitioner-Based Nationwide JAMP Study. Circulation. 2020;142:1810–20. 33131317 10.1161/CIRCULATIONAHA.120.049730 PMC7643792

[3] Kario K. What are the ideal metrics for assessing the quality of long-term stabilized “perfect” 24-h BP control after renal denervation? Hypertens Res. 2024;47:2644–51. 39191961 10.1038/s41440-024-01859-5

[4] Kario K. Nocturnal Hypertension: New Technology and Evidence. Hypertension. 2018;71:997–1009. 29712746 10.1161/HYPERTENSIONAHA.118.10971

[5] Kandzari DE, Townsend RR, Kario K, Mahfoud F, Weber MA, Schmieder RE, et al. Safety and Efficacy of Renal Denervation in Patients Taking Antihypertensive Medications. J Am Coll Cardiol. 2023;82:1809–23. 37914510 10.1016/j.jacc.2023.08.045

[6] Böhm M, Kario K, Kandzari DE, Mahfoud F, Weber MA, Schmieder RE, et al. Efficacy of catheter-based renal denervation in the absence of antihypertensive medications (SPYRAL HTN-OFF MED Pivotal): a multicentre, randomised, sham-controlled trial. Lancet. 2020;395:1444–51. 32234534 10.1016/S0140-6736(20)30554-7

[7] Kario K, Mahfoud F, Kandzari DE, Townsend RR, Weber MA, Schmieder RE, et al. Long-term reduction in morning and nighttime blood pressure after renal denervation: 36-month results from SPYRAL HTN-ON MED trial. Hypertens Res. 2023;46:280–8. 36241705 10.1038/s41440-022-01042-8 PMC9747613

[8] Böhm M, Townsend RR, Kario K, Kandzari D, Mahfoud F, Weber MA, et al. Rationale and design of two randomized sham-controlled trials of catheter-based renal denervation in subjects with uncontrolled hypertension in the absence (SPYRAL HTN-OFF MED Pivotal) and presence (SPYRAL HTN-ON MED Expansion) of antihypertensive medications: a novel approach using Bayesian design. Clin Res Cardiol. 2020;109:289–302. 32034481 10.1007/s00392-020-01595-z PMC7042193

[9] Kandzari DE, Böhm M, Mahfoud F, Townsend RR, Weber MA, Pocock S, et al. Effect of renal denervation on blood pressure in the presence of antihypertensive drugs: 6-month efficacy and safety results from the SPYRAL HTN-ON MED proof-of-concept randomised trial. Lancet. 2018;391:2346–55. 29803589 10.1016/S0140-6736(18)30951-6

[10] Townsend RR, Mahfoud F, Kandzari DE, Kario K, Pocock S, Weber MA, et al. Catheter-based renal denervation in patients with uncontrolled hypertension in the absence of antihypertensive medications (SPYRAL HTN-OFF MED): a randomised, sham-controlled, proof-of-concept trial. Lancet. 2017;390:2160–70. 28859944 10.1016/S0140-6736(17)32281-X

[11] McEvoy JW, McCarthy CP, Bruno RM, Brouwers S, Canavan MD, Ceconi C, et al. 2024 ESC Guidelines for the management of elevated blood pressure and hypertension. Eur Heart J. 2024;45:3912–4018. 39210715 10.1093/eurheartj/ehae178

[12] Kario K, Shin J, Chen CH, Buranakitjaroen P, Chia YC, Divinagracia R, et al. Expert panel consensus recommendations for ambulatory blood pressure monitoring in Asia: The HOPE Asia Network. J Clin Hypertens. 2019;21:1250–83. 10.1111/jch.13652 PMC8030405 31532913

[13] Mancia G, Verdecchia P. Clinical value of ambulatory blood pressure: evidence and limits. Circ Res. 2015;116:1034–45. 25767288 10.1161/CIRCRESAHA.116.303755

[14] Mancia G, Kreutz R, Brunström M, Burnier M, Grassi G, Januszewicz A, et al. 2023 ESH Guidelines for the management of arterial hypertension The Task Force for the management of arterial hypertension of the European Society of Hypertension: Endorsed by the International Society of Hypertension (ISH) and the European Renal Association (ERA). J Hypertens. 2023;41:1874–2071. 37345492 10.1097/HJH.0000000000003480

[15] Grassi G, Seravalle G, Quarti-Trevano F, Dell’Oro R, Bombelli M, Cuspidi C, et al. Adrenergic, metabolic, and reflex abnormalities in reverse and extreme dipper hypertensives. Hypertens. 2008;52:925–31. 10.1161/HYPERTENSIONAHA.108.116368 18779438

[16] de la Sierra A, Redon J, Banegas JR, Segura J, Parati G, Gorostidi M, et al. Prevalence and factors associated with circadian blood pressure patterns in hypertensive patients. Hypertension. 2009;53:466–72. 19171788 10.1161/HYPERTENSIONAHA.108.124008

[17] Grassi G, Bombelli M, Seravalle G, Dell’Oro R, Quarti-Trevano F. Diurnal blood pressure variation and sympathetic activity. Hypertens Res. 2010;33:381–5. 20203684 10.1038/hr.2010.26

[18] Kario K. Time for focus on morning hypertension: pitfalls of current antihypertensive medication. Am J Hypertens. 2005;18:149–51. 15752939 10.1016/j.amjhyper.2004.09.007

[19] Kario K, Tomitani N, Nishizawa M, Harada N, Kanegae H, Hoshide S. Concept, study design, and baseline blood pressure control status of the nationwide prospective HI–JAMP study using multisensor ABPM. Hypertens Res. 2023;46:357–67. 36380199 10.1038/s41440-022-01087-9

[20] Cuspidi C, Tadic M, Sala C, Carugo S, Mancia G, Grassi G. Reverse dipping and subclinical cardiac organ damage: a meta-analysis of echocardiographic studies. J Hypertens. 2021;39:1505–12. 33657585 10.1097/HJH.0000000000002836

[21] Tan X, Sundström J, Lind L, Franzon K, Kilander L, Benedict C. Reverse dipping of systolic blood pressure is associated with increased dementia risk in older men: A longitudinal study over 24 Years. Hypertension. 2021;77:1383–90. 33550821 10.1161/HYPERTENSIONAHA.120.16711

[22] Yamamoto Y, Akiguchi I, Oiwa K, Hayashi M, Ohara T, Ozasa K. The relationship between 24-hour blood pressure readings, subcortical ischemic lesions and vascular dementia. Cerebrovasc Dis. 2005;19:302–08. 15775671 10.1159/000084498

[23] Ueda T, Kawakami R, Nakada Y, Nakano T, Nakagawa H, Matsui M, et al. Differences in blood pressure riser pattern in patients with acute heart failure with reduced mid-range and preserved ejection fraction. ESC Heart Fail. 2019;6:1057–67. 31325235 10.1002/ehf2.12500 PMC6816074

[24] Kario K, Hoshide S, Mizuno H, Kabutoya T, Nishizawa M, Yoshida T, et al. Nighttime hemodynamic phenotype. A novel risk factor for cardiovascular disease, especially heart failure: the practitioner-based nationwide JAMP study. Clin Res Cardiol. 2023;112:98–110. 35760927 10.1007/s00392-022-02051-w

[25] Matsui Y, Ishikawa J, Eguchi K, Shibasaki S, Shimada K, Kario K. Maximum value of home blood pressure: a novel indicator of target organ damage in hypertension. Hypertension. 2011;57:1087–93. 21536993 10.1161/HYPERTENSIONAHA.111.171645

[26] Fujiwara T, Hoshide S, Kanegae H, Kario K. Clinical Impact of the Maximum Mean Value of Home Blood Pressure on Cardiovascular Outcomes: A Novel Indicator of Home Blood Pressure Variability. Hypertension. 2021;78:840–50. 34304579 10.1161/HYPERTENSIONAHA.121.17362

[27] Kario K, Tomitani N, Fujiwara T, Okawara Y, Kanegae H, Hoshide S. Peak home blood pressure as an earlier and strong novel risk factor for stroke: the practitioner-based nationwide J-HOP study extended. Hypertens Res. 2023;46:2113–23. 37076610 10.1038/s41440-023-01297-9 PMC10113967

[28] Kario K, Okawara Y, Kanegae H, Tomitani N, Hoshide S. Peak nocturnal home blood pressure as an early and strong novel risk factor for stroke: the practitioner-based nationwide J-HOP nocturnal BP study. Hypertens Res. 2024; 10.1038/s41440-024-01866-6. 10.1038/s41440-024-01866-6 39242824

[29] Drawz PE, Pajewski NM, Bates JT, Bello NA, Cushman WC, Dwyer JP, et al. Effect of intensive versus standard clinic-based hypertension management on ambulatory blood pressure. Hypertension. 2017;69:42–50. 27849563 10.1161/HYPERTENSIONAHA.116.08076 PMC5145774

[30] Ferrannini E, Cushman WC. Diabetes and hypertension: the bad companions. Lancet. 2012;380:601–10. 22883509 10.1016/S0140-6736(12)60987-8

[31] Schmieder RE. Renal denervation in patients with chronic kidney disease: current evidence and future perspectives. Nephrol Dial Transplant. 2023;38:1089–96. 35617138 10.1093/ndt/gfac189 PMC10157753

[32] Kario K. Obstructive sleep apnea syndrome and hypertension: ambulatory blood pressure. Hypertens Res. 2009;32:428–32. 19494815 10.1038/hr.2009.56

[33] Thomas G, Xie D, Chen HY, Anderson AH, Appel LJ, Bodana S, et al. Prevalence and prognostic significance of apparent treatment resistant hypertension in chronic kidney disease: report from the chronic renal insufficiency cohort study. Hypertension. 2016;67:387–96. 26711738 10.1161/HYPERTENSIONAHA.115.06487 PMC4713320

[34] Ott C, Mahfoud F, Mancia G, Narkiewicz K, Ruilope LM, Fahy M, et al. Renal denervation in patients with versus without chronic kidney disease: results from the Global SYMPLICITY Registry with follow-up data of 3 years. Nephrol Dial Transplant. 2022;37:304–10. 34109413 10.1093/ndt/gfab154

[35] Chen G, McAlister FA, Walker RL, Hemmelgarn BR, Campbell NRC. Cardiovascular outcomes in Framingham participants with diabetes: the importance of blood pressure. Hypertension. 2011;57:891–7. 21403089 10.1161/HYPERTENSIONAHA.110.162446 PMC3785072

[36] Mahfoud F, Mancia G, Schmieder R, Narkiewicz K, Ruilope L, Schlaich M, et al. Renal denervation in high-risk patients with hypertension. J Am Coll Cardiol. 2020;75:2879–88. 32527396 10.1016/j.jacc.2020.04.036

[37] Schmieder RE, Mahfoud F, Mancia G, Narkiewicz K, Ruilope L, Hutton DW, et al. Clinical event reductions in high-risk patients after renal denervation projected from the global SYMPLICITY registry. Eur Heart J Qual Care Clin Outcomes. 2022;9:575–82. 10.1093/ehjqcco/qcac056 PMC10495746 36057838

[38] Kario K, Hettrick DA, Prejbisz A, Januszewicz A. Obstructive Sleep Apnea–Induced Neurogenic Nocturnal Hypertension: A Potential Role of Renal Denervation? Hypertension. 2021;77:1047–60. 33641363 10.1161/HYPERTENSIONAHA.120.16378

[39] Warchol-Celinska E, Prejbisz A, Kadziela J, Florczak E, Januszewicz M, Michalowska I, et al. Renal denervation in resistant hypertension and obstructive sleep apnea: Randomized proof-of-concept phase II trial. Hypertension. 2018;72:381–90. 29941516 10.1161/HYPERTENSIONAHA.118.11180

