# Supplementary material for: Two-year nighttime blood pressure changes after radiofrequency renal denervation: pooled results from the SPYRAL HTN trials
Source: Hypertens Res. 2025 Apr 2;48(6):1951–62. doi: 10.1038/s41440-025-02186-z (PMC12137119; doi:10.1038/s41440-025-02186-z)
Supplement: Supplementary file 1 — Supplementary Material [file 41440_2025_2186_MOESM1_ESM.docx]

**Supplementary Material**

**Two-year nighttime blood pressure changes after radiofrequency renal denervation: pooled results from the SPYRAL HTN trials**

**Supplementary Table 1. Changes in number of antihypertensive drugs from 6 months through 2 years by dipper patterns**

| **Antihypertensive drugs*** | **All cohort** | **Dippers** | **Non-dippers** | **Risers** | **P-value**** |
| --- | --- | --- | --- | --- | --- |
| **6 months** | | | | | |
| No. of antihypertensive drugs | 1.55 ± 1.06 | 1.46 ± 1.00 | 1.58 ± 1.09 | 1.84 ± 1.03 |  |
| **1 year** | | | | | |
| No. of antihypertensive drugs | 1.80 ± 1.06 | 1.81 ± 1.04 | 1.69 ± 1.08 | 2.29 ± 0.09 |  |
| Change in no. of antihypertensive drugs | 0.25 ± 0.92 | 0.35 ± 0.95 | 0.12 ± 0.91 | 0.45 ± 0.80 | 0.005 |
| **2 years** | | | | | |
| No. of antihypertensive drugs | 1.92 ± 1.18 | 1.84 ± 1.14 | 1.93 ± 1.25 | 2.30 ± 0.94 |  |
| Change in no. of antihypertensive drugs | 0.36 ± 1.06 | 0.35 ± 1.12 | 0.34 ± 1.03 | 0.49 ± 1.02 | 0.20 |

*Changes in no. of antihypertensive drugs were evaluated by drug testing where available, if not, by prescribed data. **P values are comparison of change in number of antihypertensive drugs between dippers, non-dippers and reverse dippers. Note that 6 months is the first time point that patients on the SPYRAL HTN OFF MED trial could have antihypertensive pharmacotherapy prescribed.

**Supplementary Table 2. Baseline dipper patterns for systolic blood pressure and heart rate**

|  | **SBP dippers** | **SBP non-dippers** | **SBP risers** |
| --- | --- | --- | --- |
| **HR dippers** | 103 | 114 | 19 |
| **HR non-dippers** | 39 | 73 | 15 |
| **HR risers** | 8 | 11 | 4 |

Dipper status is defined by the baseline night/day SBP or HR ratio: dippers ≤ 0.9, non-dippers >0.9 to ≤1.0, and risers >1.0

HR, heart rate; SBP, systolic blood pressure

**Supplementary Table 3. Change in 24-hour heart rate in all patients and by SBP dipper status at baseline**

| **24-h Heart Rate** | **All cohort** | **Dippers** | **Non-dippers** | **Risers** | **P-value*** |
| --- | --- | --- | --- | --- | --- |
| **Baseline** | | | | | |
| 24-h heart rate | 74.5 ± 10.5 | 73.4 ± 10.4 | 75.2 ± 10.4 | 74.8 ± 10.6 | 0.31 |
| **1 Year** | | | | | |
| 24-h heart rate | 73.3 ± 10.1 | 71.5 ± 10.2 | 74.5 ± 9.7 | 74.6 ± 11.1 |  |
| Change in 24-h heart rate | -0.8 ± 6.9 | -1.7 ± 7.0 | -0.3 ± 6.7 | 0.9 ± 7.3 | 0.02 |
| **2 Years** | | | | | |
| 24-h heart rate | 72.8 ± 10.4 | 71.2 ± 9.7 | 73.6 ± 10.8 | 75.0 ± 10.3 |  |
| Change in 24-h heart rate | -1.2 ± 8.2 | -1.8 ± 7.1 | -1.3 ± 9.0 | 1.5 ± 7.7 | 0.08 |

*P values are comparison of dippers, non-dippers and reverse dippers. Heart rate change from baseline at 1 and 2 years was not significant in the non-dipper and riser groups

**Supplementary Table 4. Baseline dipper status for patients in each subgroup**

| **Dipper pattern** | **Subgroups** | | **p-value** |
| --- | --- | --- | --- |
|  | **CKD (eGFR<60)** | **Non-CKD (eGFR≥60)** | 0.28 |
| Dipper | 28.6% (6/21) | 39.5% (144/365) |  |
| Non-Dipper | 57.1% (12/21) | 51.0% (186/365) |  |
| Riser | 14.3% (3/21) | 9.6% (35/365) |  |
|  | **OSA** | **Non-OSA** | 0.63 |
| Dipper | 36.8% (14/38) | 39.1% (136/348) |  |
| Non-Dipper | 50.0% (19/38) | 51.4% (179/348) |  |
| Riser | 13.2% (5/38) | 9.5% (33/348) |  |
|  | **T2DM** | **Non-T2DM** | 0.82 |
| Dipper | 36.7% (11/30) | 39.0% (139/356) |  |
| Non-Dipper | 53.3% (16/30) | 51.1% (182/356) |  |
| Riser | 10.0% (3/30) | 9.8% (35/356) |  |
|  | **Age <65 y** | **Age ≥65 y** | 0.64 |
| Dipper | 37.8% (125/331) | 45.5% (25/55) |  |
| Non-Dipper | 53.2% (176/331) | 40.0% (22/55) |  |
| Riser | 9.1% (30/331) | 14.6% (8/55) |  |

P values were calculated using CMH (Cochran-Mantel-Haenszel) test with modified Ridit score.

CKD, chronic kidney disease; eGFR, estimated glomerular filtration rate; OSA, obstructive sleep apnea; T2DM, type 2 diabetes mellitus


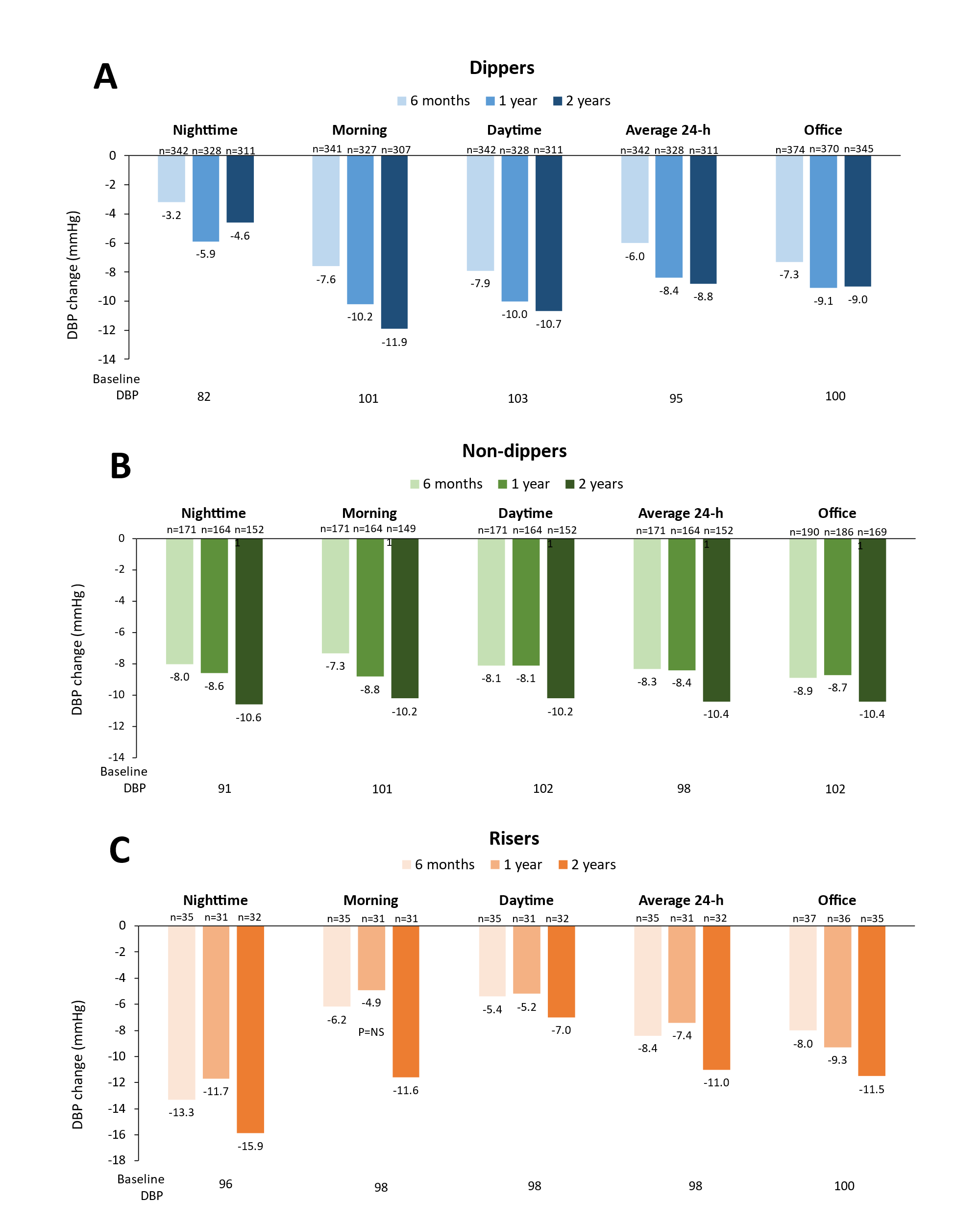


**Supplementary Figure 1. Mean diastolic blood pressure changes through 2 years after radiofrequency RDN by baseline dipping patterns.**

There was generally a progressive reduction in mean diastolic BP through 2 years follow-up, at all times of the day. A) dippers, B) non-dippers, C) risers. All BP changes compared to baseline were statistically significant (*p* < 0.05) except the 1-year change from baseline for risers in the morning. DBP, diastolic blood pressure; RDN, renal denervation
